# Re-thinking funding success in Alzheimer’s disease research: Why good science is not enough

**DOI:** 10.1016/j.tjpad.2026.100496

**Published:** 2026-01-30

**Authors:** Peter Fusdahl, Miguel G. Borda, Dag Aarsland

**Affiliations:** a Centre for Age-Related Medicine (SESAM), Stavanger University Hospital, Stavanger, Norway; b Department of Clinical Science, Faculty of Medicine, University of Bergen, Stavanger, Norway; c Department of Neurology, Clínica Universidad de Navarra, Pamplona, Spain; d Department of Old Age Psychiatry, Institute of Psychiatry, Psychology, and Neuroscience, King’s College London, London, UK

To the Editor

Leading researchers assume that sufficiently high scientific quality should lead to research funding [1]. This reflects how scientists are trained, how peer review is justified, and how success stories are told [2]. Yet experience from Alzheimer’s disease research funding suggests something else: high-quality proposals are by no means guaranteed funding – not because decisions are misguided or unfair, but because funding systems operate under structural uncertainty.

A useful way to think about funding success can be to treat it as a probability rather than a binomial verdict—a descriptive model of how funding systems function:

P(fundingsuccess)=(α+β)(timing+luck)

Here, α represents scientific quality: rigor, feasibility, impact, competence, and team track record. β reflects alignment with funding system priorities, such as policy signals, thematic calls, and institutional agendas. These are the two dimensions researchers most often focus on when proposals fail.

Neither dimension is fixed. Alignment is not static: shifts in political priorities, public narratives, or institutional strategy can rapidly redefine what counts as “relevant” or “timely” research, even when scientific quality remains constant. Moreover, quality itself is not an objective constant. It is assessed through peer review, a process shaped by norms, bias, time constraints, and reviewer heterogeneity.

What is often underestimated is the second part of the equation. Timing refers to when a proposal enters the funding system: funding cycles, policy windows, budget constraints, strategic momentum, and the temporal competitive field. Luck reflects factors largely outside the applicant’s control, including reviewer background, panel composition, and internal deliberations – rarely visible to applicants, yet sometimes a decisive factor.

Research funding involves decision-making under incomplete information, making outcomes easy to misinterpret. Rejection is often treated as evidence of insufficient quality, while success is taken as confirmation that both the science and the process were excellent. This tendency evaluating decisions by outcomes rather than by the quality of reasoning – is known as *resulting* [3] and leads to systematic mislearning under uncertainty.

Empirical evidence illustrates this clearly. In Norway, public debate was triggered when a researcher received rejection from the Research Council of Norway, only to secure substantial funding from the European Research Council later with essentially the same proposal [4]. Such cases show how assessments of “quality” can differ sharply across evaluative contexts, even among highly respected funders. Similarly, in an international survey, fewer than half of dementia researchers reported regular interaction with policymakers or public research funding decision-makers, and such engagement was more strongly associated with seniority than with project-level characteristics [1]. Together, these findings point to structural information asymmetries that shape funding outcomes alongside scientific quality.

Funding processes therefore resemble poker more than chess in key respects. Relevant information remains hidden, chance intervenes, and even optimal decisions can fail. Unlike chess, outcomes are not a simple function of skill, but of skill interacting with uncertainty.

Separating the quality of funding decisions from luck is difficult, yet hindsight bias almost always invites us to try [5]. As Daniel Kahneman observed, small and often invisible contingencies can decisively alter outcomes.

For researchers, this challenges the assumption that scientific quality alone should be enough. Excellence remains essential, but it is not sufficient in a probabilistic system. Interpreting funding decisions solely through standardized feedback risks over-correcting strong proposals after rejection and under-correcting weak ones after success. A more realistic strategy combines rigorous science with awareness of evaluator behavior, bias, noise, and timing.

For policymakers and funding institutions, the implication runs in the opposite direction. If luck and hidden information play a substantial role, observed success cannot be used uncritically as a proxy for decision quality or system performance. Structured evaluation frameworks, including emerging AI-assisted review systems, may reduce noise in reviewer assessments and improve decision consistency. Persistent concentration of funding among already successful actors may reflect cumulative advantage rather than consistently superior project selection [2]. Greater transparency about uncertainty and clearer communication of priorities may improve allocative efficiency and trust [1].

These considerations would matter in any field, but particularly in chronically underfunded Alzheimer's and dementia disease research. After four decades of effort, we still lack effective treatment or cure. Progress has been incremental and failure rates remain high [1].

Misinterpreting funding outcomes as reliable signals of merit carries real costs. It risks discouraging novel ideas and talented researchers, reinforcing conservative project selection, and narrowing the range of approaches explored in a field that can least afford it.

In dementia research, where uncertainty is structural and success remains more rare, scientific quality alone cannot explain outcomes. Recognizing funding decisions as probabilistic rather than purely judgmental is essential for learning accurately from both success and failure. In a field where patients and families continue to wait for effective prevention and treatment, improving how we interpret – and design – funding decisions is not a matter of blame, but of progress.

## Declaration of Generative AI and AI-assisted technologies in the writing process

During the preparation of this work the first author used Anthropic/Claude to improve the readability of the manuscript. After using this tool, the authors reviewed and edited the content as needed and take full responsibility for the content of the published article.

## CRediT authorship contribution statement

**Peter Fusdahl:** Conceptualization, Investigation, Methodology, Project administration, Writing – original draft, Writing – review & editing. **Miguel G. Borda:** Writing – review & editing. **Dag Aarsland:** Writing – review & editing.

## Declaration of competing interest

Dag Aarsland reports a relationship with Journal of Prevention of Alzheimer’s Disease that includes: board membership. The authors declare that they have no other known competing financial interests or personal relationships that could have appeared to influence the work reported in this paper.

## References

[1] Fusdahl P., Borda M.G., Baldera J.P., Aarsland D., Khachaturian A., Braut GS. (2024). Perspectives of old-age and dementia researchers on communication with policymakers and public research funding decision-makers: an international cross-sectional survey. Front Med.

[2] Bol T., de Vaan M., van de Rijt A. (2018). Proceedings of the National Academy of Sciences.

[3] Duke A. (2019).

[4] Khrono.no (2025). https://www.khrono.no/er-jeg-toppforsker-bunnforsker-eller-begge-deler/980616?utm_source=chatgpt.com.

[5] Kahneman D. Thinking, fast and slow: macmillan; 2011.

